# Influence of Anticorrosive Surface Treatment of Steel Reinforcement Fibers on the Properties of Ultra-High Performance Cement Composite

**DOI:** 10.3390/ma15238401

**Published:** 2022-11-25

**Authors:** Lubos Bocian, Radoslav Novotny, Frantisek Soukal, Jakub Palovcik, Matej Brezina, Jan Koplik

**Affiliations:** Faculty of Chemistry, Brno University of Technology, Purkynova 118, 612 00 Brno, Czech Republic

**Keywords:** ultra-high-performance concrete, reactive powder concrete, flexural strength, compressive strength, brass

## Abstract

The influence on the bond between the steel fiber and the matrix of the anticorrosive treatments of steel used for concrete reinforcement is not yet fully understood. The topic of steel fiber treatment was not also studied clearly in terms of brass removal before. This paper deals with how the brass on the surface of steel fibers behaves in the UHPC matrix and how it affects its properties. The steel fibers were firstly modified with a number of surface treatments to remove brass on their surface. Some of the treatments have never been tried before for this purpose. Secondly, the surface of the fibers was analyzed by SEM, EDS, XRF, and stereomicroscopy. Lastly, the properties of the composites were analyzed. It was found out that the majority of brass on the surface of the fibers could be removed by mixture of NH_3_ and H_2_O_2_ with a ratio of 3:1 (*v*/*v*). It was also found out that the surface treatment slightly affects the mechanical properties, but it does that only by mechanical interlocking between the fiber and the matrix. No dissolution of the surface treatment was observed under the given conditions. According to the results, steel fibers without surface treatment should be used in UHPC if available.

## 1. Introduction

The emergence of UHPC has sprung up new possibilities of concrete usage which have been made possible by a unique set of properties like high compressive strength and durability [1]. These can be achieved by optimization of the granular mixture, low w/c ratio and use of superplasticizer. Ductility and high flexural strength can be achieved by blending steel fibers into the UHPC [2,3]. Fibers of varying sizes can be used [4,5,6,7]. Fiber composites can be used in applications such as impact protection [8].

There are two failure modes for fibers in fiber reinforced UHPC. The first mode occurs when the bond strength is below the tensile strength of the fiber. This leads to fiber being pulled out of the matrix. The second failure mode occurs when the bond strength is greater than the tensile strength of fiber [9].

Three mechanisms are involved in the interfacial bonding. The first one is physicochemical adhesion. The second mechanism is friction between the fiber and the matrix. Both of those mechanisms act at the fiber-matrix interface. The third mechanism is mechanical interlocking due to the morphology of the fiber surface. The bond strength between the plain steel fiber and the CSH gel is governed mainly by the density of the interface [9,10]. There are some ways to improve the interfacial zone. These methods range from modifying matrix properties such as water/cement ratio. However, there are methods for modifying steel fibers that range from mechanical modification of the surface or chemical treatment to deposition of nano-SiO_2_ on the surface of steel fibers. Note that direct measurement of bond strength is achieved by pull-out tests, but the lack of one standard makes them difficult to compare between researchers. The measurement of flexural and compressive strength can serve as an indirect method of measurement of bond strength, and it is also more comparable due to existing standards. Most of the research lack data regarding optical microscopy of treated fibers [9,11,12,13].

The steel fibers used in UHPC are made by drawing a series of dies made from very hard materials. Lubricants are used for dry wire drawing, but they are removed from the surface of a fiber before any coating operation. One of the notable applications of steel fibers includes its usage as a tire bead, in which case the wire is brass plated [14,15]. Brass plating on the surface of steel fiber increases the bond strength between the steel fiber and the rubber compound of a tire. There are a couple of methods for plating steel fibers. The usual brass plating consists of 65–68% copper. However, it should be noted that uncoated steel fibers can be purchased right now [16,17,18].

Steel passivates in the basic environment of concrete, but it can corrode in some situations. One method to prevent this is hot-dip galvanization. The upper layer of the hot-dip galvanized surface consists of pure Zn with minor impurities. There are concerns about the behavior of Zn in concrete. The main area of dispute is its possible corrosion and hydrogen evolution, both of which are pH dependent [10,19].

Pokorny et al., found out that the porosity is always higher in concrete specimens with reinforcement with hot-dip galvanized layer [10,19]. From the works of Macias et al., and Blanco et al., can be concluded that the dissolution and hydrogen evolution of the hot-dip galvanized layer depends on pH, while the type of corrosion products depends on the concentration of Ca^2+^ in the solution [20,21,22]. Tashiro et al., studied bond strength between steel, copper, and hot-dip galvanized wire in C_3_S. Steel wire showed highest bond strength, while hot-dip galvanized wire showed the lowest of all of them. The calcium hydroxyzincate layer was observed between the hot-dip galvanized wire and the matrix. Both Zn and Cu tended to migrate to matrix via diffusion [23].

There is also some research on the bond strength of brass coated steel fibers in UHPC. Pi et al., studied the bond strength and corrosion of steel fibers in UHPC. They found out that the brass coated surface is smooth. They concluded that the pullout leads to the removal of brass on the surface of the fiber. It was also found out that the matrix could potentially dezincificate brass [24]. Corinaldesi et al., found out that the addition of burnt lime to UHPC increases flexural strength of specimens with brass and Zn coated steel fibers. The increase in flexural strength is attributed to the formation of calcium hydroxyzincate. It is also theorized that similar compounds could form in case of copper [25]. Novotny et al., noted an increase of flexural strength of UHPC with the addition of burnt lime. However, this occurred in the case of matrix without fibers [26]. Citek et al., found out that the brass coating is beneficial in UHPC matrix while decreasing bond strength in normal strength concrete. The composition of the matrix is not stated by the authors though [27].

Chun et al., observed that sanding the surface of a fiber along its axis and in a perpendicular direction to its axis increases surface roughness of steel fibers and bond strength. It seems quite unrealistic to modify the fiber one by one for practical use though [11]. Chun et al., also studied effects of some chemical treatments on bond strength. The acetone bath, based on previous research, increased the surface roughness of the steel fiber surface according to AFM. However, this was not confirmed by SEM. Authors also claim that the acetone removes debris from the surface. They found out that acetone bath significantly increases bond strength [12,28]. Chun et al., also discovered that bond strength is significantly increased by HCl bath, nanosilica deposition, and EDTA treatment [12].

Yoo et al., discovered that corrosion in 3.5% solution of NaCl can remove the brass coating from the surface and reveal the surface beneath it. Corroded steel fiber with corrosion products mechanically removed showed significantly improved bond strength [29,30]. Kim et al., Yoo et al., and Chun et al., studied corrosion of brass-coated steel fibers in the presence of EDTA. They found out that the corrosion in the presence of EDTA leads to a much coarser surface and the removal of brass. It also improves bond strength. Chun et al., warned that prolonged corrosion could lead to weakening of the fibers [12,31,32].

It is also necessary to review the chemical and corrosion properties of the metals present in this study because they were not properly reviewed in previous research. Iron is dissolved under acidic conditions and a pH value below 9. It is passivated in presence of oxidizing acids such as conc. HNO_3_ and dissolved when the pH value exceeds value of 13. The most commonly used surface treatment methods against corrosion are phosphating and passivation in hydroxides [33,34,35,36]. Copper is not dissolved in non-oxidizing acids without oxidizing agents. It dissolves in dilute HNO_3_ and hot concentrated H_2_SO_4_. It is generally very vulnerable to chemical attack by oxidizing and complexing species. It readily forms complexes with both H_2_O and NH_3_. Copper is inactive within pH values from 7 to 13 [33,34,35,36]. Zinc readily dissolves both in acidic and basic environments. It has amphoteric properties. It forms stable complexes with H_2_O and NH_3_ [34,35,36]. Dezincification of brass is also a rather important phenomenon to understand when talking about corrosion of brass with more than 15% zinc. It is selective corrosion and dissolution of zinc from brass that leads to the destruction of brass as a whole. When the brass dezincificates, the Zn is dissolved, but minor dissolution and redeposition of Cu can also be observed. The Cu left after the dezincification process has spongy-like structure [37].

In this study, we firstly employ some of the chemical treatments based on literature [12] and try to improve mechanical treatment based on literature for practical use while proposing our own chemical treatments that have never been used before for the purpose of brass removal [11]. We analyze the composition of brass that has been left on the fiber surface by XRF and study the morphology by stereomicroscopy. We also analyze surface treatment of selected chemically treated fibers by SEM and EDS. Finally, we delve into the determination of mechanical and other properties of prepared composites and study the interaction between brass and the UHPC matrix by SEM. The study is based on the master thesis of the same name made by author of this paper [38].

## 2. Materials and Methods

### 2.1. Fiber Surface Treatment Methods

The first chemical treatment methods were conducted in 1000 mL beakers with (15 ± 0.5) g of steel fibers 12.5 × 0.2 mm (KrampeHarex, D, Hamm, Germay) except for the mechanical treatment method. The fibers were all immersed in approximately 100–150 mL of solution. Only slight mechanical agitation was used throughout all experiments. The experiment was observed for changes in color or evolution of any gas. The experiments were ended when there was no change in color or no visible gas evolution. The fibers were then filtrated in Büchner’s funnel and rinsed with demineralized water. The fibers were then dried in a preheated oven at 120 °C for 1 h. All experiment durations of experiments that used chemicals and their concentrations are displayed in Table 1. The suppliers, origin and qualities of the chemicals can be seen in Table 2. Chemical treatments were chosen based on the literature mentioned in the introduction.

For mechanical surface treatment, 100 g of fibers were put in a GFL 3040 turnover mixer in a 1.5 L PET bottle with 200 g of mesh 40 synthetic corundum. The rotational speed was 12 RPM. The fibers were left in the turnover mixer for 28 h. The fibers were then separated by magnet from synthetic corundum.

In case of composite fabrication, the 1–1.2 kg of fibers were chemically treated in 5 L PP beaker. The fibers were fully immersed in the solution. Approximately 2 L of solution were used every time. The fibers were then decanted five times and dried in a preheated oven for 1 h at 160 °C. Mechanically treated fibers for composite fabrication were made by the same method as described above. The only change was in the overall quantity of them (approximately 1 kg). All fibers were then stored in 5l buckets with silica gel in bags until the fabrication of the composites. 

### 2.2. Composite Fabrication

The composition of the mixture composition can be seen in Table 3. This mixture composition was found out to have properties best properties in terms of other ongoing and still unpublished research on our university at the time. The potassium formate was synthesized from KOH (technical quality 99%, Fichema, Brno-Líšeň, Czech Republic) and Ca (HCOO)_2_ (technical quality 98%, CHEMlogistics, Pardubice, Czech Republic). Every powder except micronized sands and steel fibers were weighted into a mixer bowl. Steel fibers and micronized sands were placed into separate bowls. The first 248 mL of demineralized water was measured in a 250 mL graduated cylinder. The 45 mL of superplasticizer and chemicals were added into the cylinder also and mixed by tipping the cylinder over.

The powders in the mixer bowl were then homogenized with a planetary mixer (KitchenAid) for 1 min at the lowest possible mixing rate. The contents of the cylinder were then added to the mixture and the mixture was mixed until plastification. The micronized sands were added after plastification. The mixing rate was set to the highest setting after plastification. Another 30 mL of water was added from the cylinder after 5 min. Water was dosed into the cylinder with a 25 mL syringe. The steel fibers were slowly added into the mixture after 8 min. The mixing was stopped at 10 min and the mini-cone slump flow test was done 30 s after dosing the composite into the cone.

**Figure 1:**
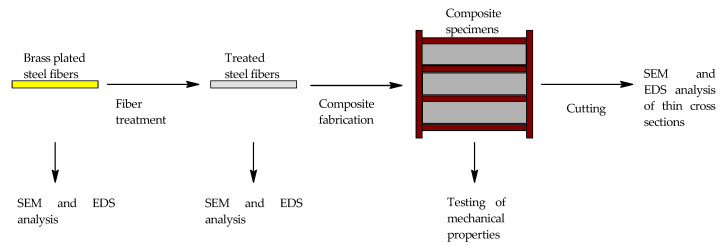
Whole experimental scheme.

The fresh mixture was then degassed in a vacuum chamber with an agitator. The degassing was considered complete when boiling of water was observed. The composite was then quickly poured into the molds. The composites were poured into the molds perpendicularly to the length of the sample. This leads to most of the fibers being preferably oriented along the length of the specimens. No special technique was employed to ensure an even distribution of the fibers. The molds were then vibrated for 30 s, and 18 specimens with dimensions of 40 × 40 × 160 mm of reference composite, composite with fibers from NH_3_/H_2_O_2_, composite with fibers from HCl, composite with fibers from HNO_3_, and composite with mechanically treated fibers were made. The specimens were demolded after 24 h, and the testing of mechanical properties was done after 24 h, 7 days, 28 days, and 95 days. The specimens for mechanical properties testing after 7 days, 28 days and 95 days were stored under water with average temperature of 18 °C. The whole experimental scheme can be seen in Figure 1.

### 2.3. Analytical Methods

X-ray fluorescence spectrometry was conducted on an Olympus Vanta VCR handheld device. This analytical method was chosen due to time considerations with a large amount of chemical treatments and time needed for the SEM and EDS analysis, although it is not perfect method for this type of specimens.

A Zeiss Stemi 2000-C stereomicroscope was used for optical analysis of the fiber surface.

Zeiss EVO LS10 SEM with X-Max 20 (Oxford Instruments, Abingdon, UK) EDS was used for analysis of surface of fibers. The fibers were analyzed at 15 kV of accelerating voltage.

JEOL JSM-7600F SEM with Ultim Max 100 (Oxford Instruments, Abingdon, UK) EDS was used for the analysis of the fiber-matrix interfacial zone of the reference composite and composite with fibers treated with NH_3_/H_2_O_2._ Cross sections were analyzed at 5 kV accelerating voltage and observed via backscattered electrons. This SEM was also used for the SEM imaging and EDS mapping of untreated steel fiber that was analyzed.

### 2.4. Mechanical Properties Testing

Flexural strength was determined by universal testing machine Instron 5985 with 250 kN load cell. The span of supports for testing were 100 mm. The load rate was 3 mm/min until the load did not reach the value of 5 kN. Then, the load rate was 0.08 kN/s. Specimens of the above-mentioned dimensions were used. Three specimens were used for flexural strength testing after 24 h, 7 days, and 95 days. Five specimens were used for flexural strength testing after 28 days. Flexural strength tests were carried out according to the CSN EN ISO 196-1 standard.

Compressive strength was determined on concrete testing machine from Czech brand BetonSystem with 3 MN load cell. The loaded area measured 1600 mm^2^ and the loading rate was 2.4 kN/s. Specimens of the above-mentioned dimensions were used after the flexural strength test and the compressive strength was measured on both sides of the specimens. Three specimens were used for compressive strength testing after 24 h, 7 days, and 95 days. Five specimens were used for compressive strength testing after 28 days. Compressive strength tests were carried out according to the CSN EN ISO 196-1 standard.

## 3. Results and Discussion

### 3.1. Fiber Surface Treatment

#### 3.1.1. X-ray Fluorescence Spectrometry

As stated above, the XRF was chosen mainly because of the large number of samples at the beginning (almost 40). The results of XRF can be seen in Table 4. The results are ordered in terms of removal of Zn from the best to the worst. It can be seen from the table that the best overall chemical treatment method for brass removal is NH_3_/H_2_O_2_, followed by HNO_3_. The third best method in terms of Zn removal is the method using (NH_4_)_2_CO_3_. This method could potentially lead to better results if the treatment time were longer. The fourth best method in terms of Zn removal is the method that involves NH_4_Cl. It could also potentially lead to better results if the treatment time was longer, but there were red stains throughout the duration of the treatment on the surface of the solution that could possibly be formed by Fe (OH)_3_ indicating that this solution corroded the steel which is a probable explanation if the solution contains chlorides [39]. The third worst method in terms of Zn removal is the chemical treatment with HCl. However, this treatment was done mainly to see if dezincification could potentially lead to removal of brass on itself. The second worst in terms of Zn removal is H_2_SO_4_. It is also worth noting that it did not lead to removal of most of the Cu, but the fact that it occurred could possibly indicate that the removal of brass through dezincification could be realistic although slow and difficult. However, this could be solved by adding an oxidizing agent into the solution [33]. Notice that fibers treated with H_2_SO_4_ show far more sulfur when analyzed by XRF. This could be due to sulfates left on the surface. The worst treatment in terms of Zn removal is mechanical treatment, which could potentially lead to removal of brass if the fibers were treated for a much longer time.

#### 3.1.2. Optical Microscopy

**Figure 2:**
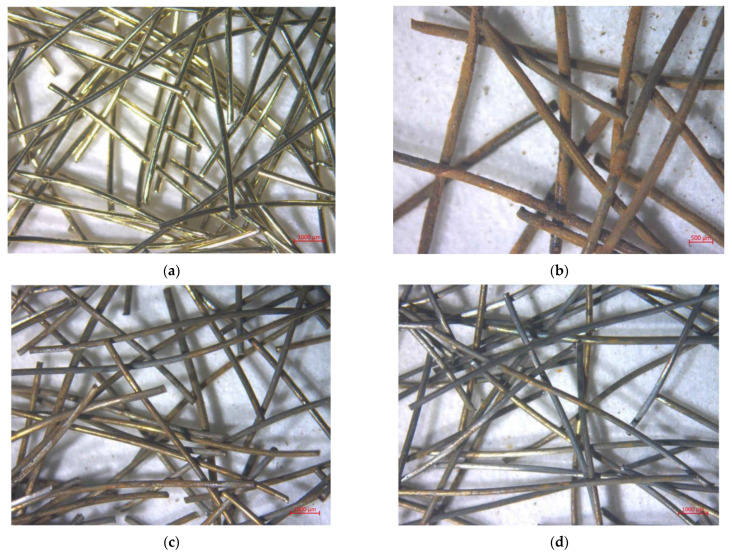
First set of mages from optical microscopy of chemically treated fibers (**a**) untreated (**b**) H_2_SO_4_ (**c**) NH_4_Cl (**d**) (NH_4_)_2_CO_3_.

The first set of images of fibers from selected chemical treatments from optical microscopy can be seen in Figure 2. The image of brass-coated fibers from optical microscope can be seen in Figure 2a. They are gold in color and very shiny. In Figure 2b we can see the fibers treated in H_2_SO_4_. The most notable thing about them is the rust, which covers most of their surface. Relatively high amount of rust compared to other treatments could be caused by sulfates that were left on the surface of the fibers, so rusting could occur at higher rate while drying in an oven. Figure 2c shows the image of fibers treated in a concentration of NH_4_Cl and NH_3_ solution of 100 g/L. Rust and some of the brass on them is clearly visible. In Figure 2d, we can see fibers treated in solution of (NH_4_)_2_CO_3_. It is worth noting that even though there is some visible rust, the fibers clearly are not as rusty as fibers from previously discussed treatments. Shiny bare steel is clearly visible on them in some places and the brass is limited to a few spots on the fibers.

**Figure 3:**
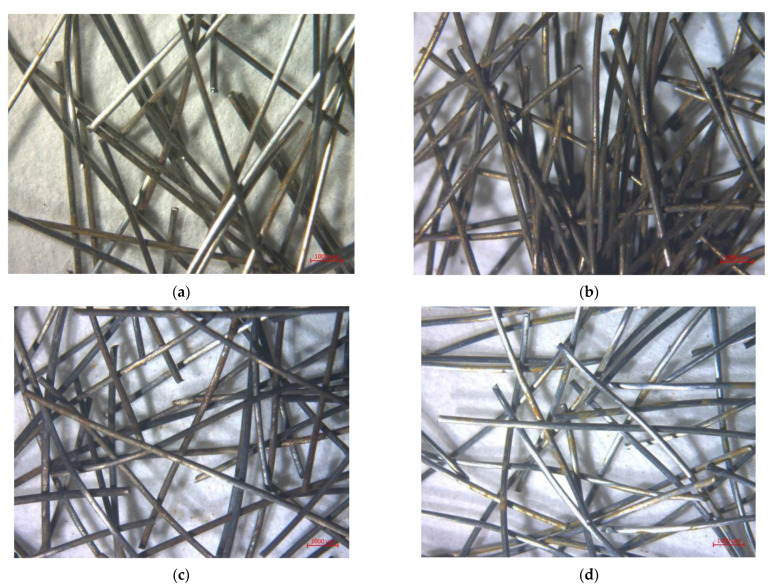
First set of mages from optical microscopy of chemically treated fibers (**a**) Mechanical treatment (**b**) HCl (**c**) HNO_3_ (**d**) NH_3_/H_2_O_2_.

The second set of fiber images from selected chemical treatments by optical microscopy can be seen in Figure 3. In Figure 3a, we can see mechanically treated steel fibers. The most interesting thing in this case is that the fibers look like bare steel. This is most likely to be the product of unintentionally polishing the brass on the fibers because it did not lead to the removal of brass as provided by the information above and below. Note that rust is not the product of the treatment, but it occurred throughout long-term storage. Fibers treated in HCl are shown in Figure 3b. The first noticeable thing is the amount of rust that built up on the fibers throughout drying in an oven. Some brass can still be clearly seen on the fibers. Fibers treated in HNO_3_ (Figure 3c) contain visible rust at the surface, but the product that is black in color is more prevalent. It is believed that the black product is Fe_3_O_4_. However, it should be noted that the fibers used for composite fabrication also had red product on them directly after drying, so the neutralization with hydroxide to prevent rust from occurring is relatively effective in only a small quantity. However, this could be solved by either quicker neutralization or using fuming HNO_3_ so that HNO_3_ hopefully would not dilute to less than 68%. The fibers treated with NH_3_/H_2_O_2_ can be seen in Figure 3d. The shiny bare steel is clearly visible on the fibers. There are some localized rust products that are reddish orange that could potentially be products of drying or long-term storage.

#### 3.1.3. SEM and EDS Analysis of the Surface of the Fibers

**Figure 4:**
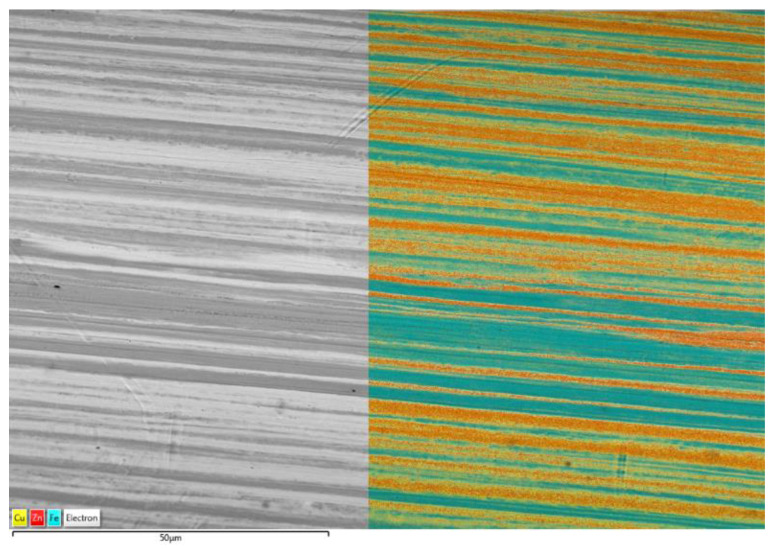
SEM image and EDS mapping of the brass coated steel fiber surface.

In Figure 4 we can see the SEM and EDS mapping of the untreated brass-coated steel fiber. Figure 4 shows the SEM image and EDS mapping of the surface of brass-coated steel fiber. It can be seen in the image that the surface is smooth with only some minor imperfections. The brass on the surface of the fiber is made of filament-like structures on the surface. The EDS map shows a very irregular elemental composition over the fiber surface. There are areas where there is majority of either Fe, Cu, or Zn every time. This is due to the uneven surface coating of brass on the surface of the steel fibers.

**Figure 5:**
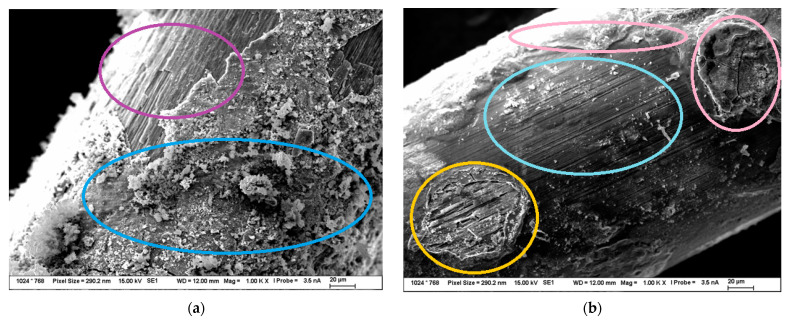

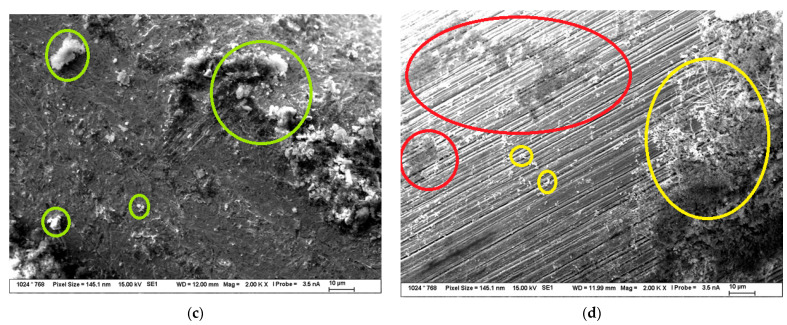
SEM images of the fiber surface used for composite fabrication (**a**) HNO_3_ (**b**) HCl (**c**) Mechanical treatment (**d**) NH_3_/H_2_O_2_.

In Figure 5 there are SEM images of the surfaces of treated fibers used for composite fabrication. The fiber treated in conc. HNO_3_ (Figure 5a) has a rough and damaged surface (purple circle) and new corrosion products (blue circle) on it. This surface was put through EDS analysis, and it was found out that Cu, O, and Fe can be found there. This indicates that there is still some Cu left on the surface and possibly indicates that there will always be some rust on the surface when oxidizing acids are used.

The SEM image of the surface of steel fiber treated in HCl can be seen in Figure 5b. Some grooves can be clearly seen in the center of the image (turquoise circle). This indicates that there are grooves on the original surface of the steel fiber that can be possibly uncovered, although there is still some brass left on the fibers. The formation to the left of the image could indicate this as there are grooves on the fiber (orange circle), but the EDS analysis indicated Cu and Zn. However, this changes when speaking of the formations in the image that can be seen on the upper part and the right part (pink circles) where it was found out by EDS analysis that the constituents are only Fe and O, which indicates that those formations are only made of rust products present on the fiber.

In Figure 5c, the surface of the mechanically treated fiber is shown. The surface is much rougher than the surface of the brass-coated steel fiber. There was some Al and Ti possibly from the synthetic corundum detected by EDS analysis on surface formations (green circles). It is relatively interesting to see that this treatment did not really do much in terms of brass removal, but it polished the surface of the fibers, although the surface is not smooth after the treatment.

SEM image of fibers treated in NH_3_/H_2_O_2_ can be seen in Figure 5d. The grooves on the original surface of the steel fiber can be clearly seen in the image. They look deeper than the grooves on the surface of the fiber treated in HCl, and they are likely to contribute to the enhanced mechanical properties of the composites. The EDS analysis was performed on the darker spots (red circles) in the upper part of the SEM image, and it was found that they are composed of Cu. The formations in the right part of the SEM image (yellow circles) and the formations that are found throughout the image were put through the EDS analysis and it was found out that they are composed of Fe and O, so they are possibly steel corrosion products.

### 3.2. Composites with Surface-Treated Fibers

#### 3.2.1. Slump Flow of Fresh Mixture

The average results of the mini-cone slump flow tests can be seen in Table 5. The acceptable slump flow for processing our type of composite is supposed to be equal to or higher than 160 mm. All composites fabricated with treated fibers had an acceptable slump flow which can be seen in Table 4 so there is probably no significant effect of fiber treatment on it. The only negative effect on slump flow could be due to the corrosion products not adhering to the surface and their dispersion into the matrix. Something similar could also be true in the case of composites with mechanically treated fibers that had metal dust present on them, and there could also be some corundum still left.

#### 3.2.2. SEM and EDS Analysis of the Fiber-Matrix Interfacial Zone

**Figure 6:**
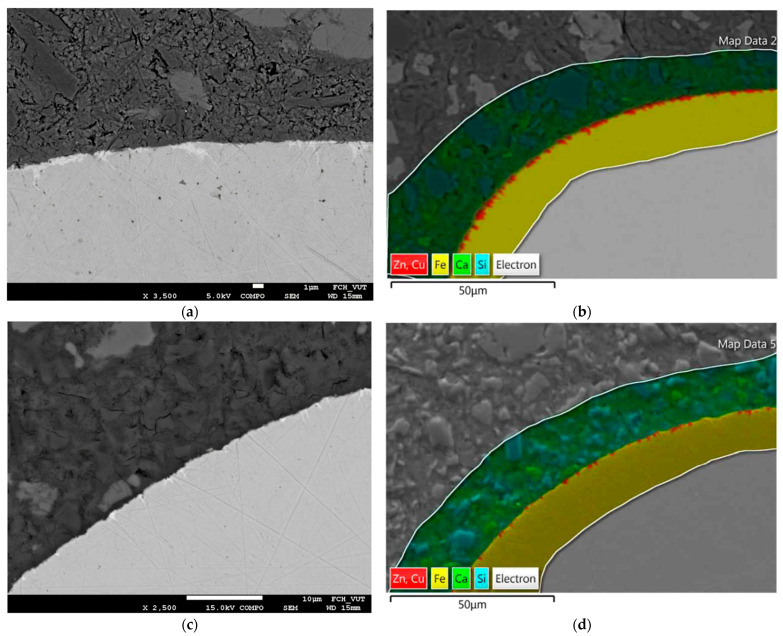
BSEM images and EDS analysis of the fiber-matrix interface: (**a**) BSEM image of the fiber-matrix interface of the composite with brass coated fibers; (**b**) EDS analysis of the fiber-matrix interface of the composite with brass coated fibers; (**c**) BSEM image of the fiber-matrix interface of the composite with fibers treated in NH_3_/H_2_O_2_; (**d**) EDS analysis of the fiber-matrix interface of the composite with fibers treated in NH_3_/H_2_O_2_.

In Figure 6, the BSEM images and the EDS analyses of fiber-matrix interface are presented. Figure 6a shows the BSEM image of the fiber matrix interfacial zone of the composite with untreated (brass coated) fibers. It can be seen that the brass fills the grooves and voids on the fiber surface and the brass layer is not continuous. It does not cover all of the fiber surface. The voids filled with brass could be quite deep somewhere reaching even 5 microns into the fiber. It should be also noted that the fiber-matrix interface is relatively compact and there is no observed additional porosity of corrosion products. Figure 6b shows the EDS analysis of the fiber-matrix interfacial zone of composite with brass coated fibers. It can be clearly seen that no observed dissolution of Zn and Cu into the matrix is observed. Figure 6c shows the BSEM image of the fiber-matrix interfacial zone of the composite with fibers treated with NH_3_/H_2_O_2_. It can be clearly seen that there are still some remains of brass on the surface of the fiber. It should be noted that the matrix fills the grooves of the steel fiber which leads to mechanical interlocking between the fiber and matrix. Figure 6d shows the EDS analysis of the fiber matrix interfacial zone of composite with fibers treated in NH_3_/H_2_O_2._ It confirms again that there are still some remains of brass left on the fiber, but there is no observed dissolution of Zn and Cu into the composite matrix.

#### 3.2.3. Mechanical Properties of Composites

**Figure 7:**
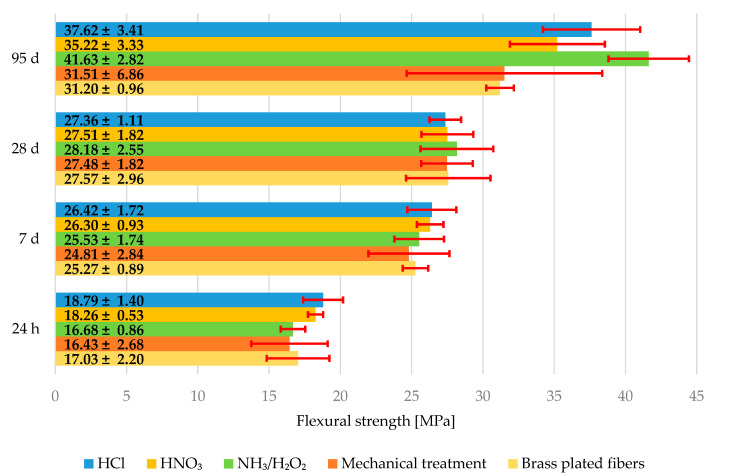
Average flexural strength of the specimens after 24 h, 7, 28, and 95 days.

The average flexural strengths are shown in graph in Figure 7. Error bars represent measurement standard deviation of the measurement. The flexural strengths after 24 h, 7, and 28 days are statistically almost of the same value and the changes could be contributed to minor errors during composite fabrication, although the composite with fibers treated with NH_3_/H_2_O_2_ shows a little bit higher flexural strength at 28 days than the composite with untreated steel fibers. The flexural strength of the composite with untreated steel fibers is (27.57 ± 2.96) MPa and (28.18 ± 2.55) MPa for the composite with fiber treated with NH_3_/H_2_O_2_ at 28 days. The difference is greater in the case of flexural strength after 95 days. The flexural strength of composite with untreated steel fibers is (31.20 ± 0.96) MPa and (41.63 ± 2.82) MPa for the composite with fibers treated with NH_3_/H_2_O_2_ at 95 days. This strength improvement between 28 and 95 days can be explained by the grooves on the fibers without brass coating and the type of cement used. Aalborg white cement contains a higher amount of C_2_S, so the improvement due to hydration and CSH gel growth can be expected in long-term mechanical properties such as flexural strength at 95 days.

**Figure 8:**
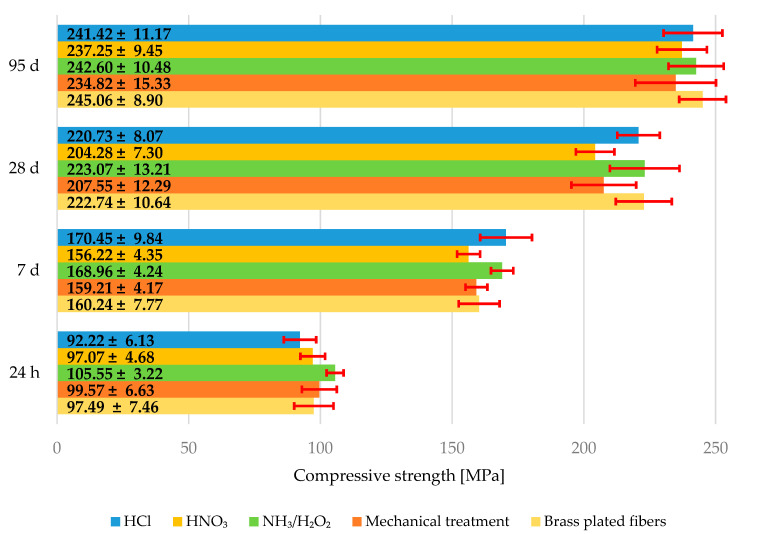
Average compressive strength of specimens after 24 h, 7, 28, and 95 days.

The average values of compressive strength can be seen in graph in Figure 8. The error bars represent the standard deviation. There is a slight reduction in the compressive strength of composites with HNO_3_ treated fibers and composites with mechanically treated fibers at every age. The values of compressive strength for composites with brass plated fibers, composites with fibers treated in HCl, and composites with fibers treated in NH_3_/H_2_O_2_ are almost the same at every age. However, this indicates that the grooves in the fibers do not contribute very much to compressive strength, although the addition of fibers alone can significantly increase the compressive strength of UHPC [6].

It is quite interesting that the composites with mechanically treated fibers have almost the same flexural strength as composites with brass plated fibers most of the time, although the standard deviation has higher value than that of flexural strengths of composites with brass-plated fibers. This could be due to two things. There is some synthetic corundum left between the fibers that served as a defect in the matrix. However, there was also some metal dust left on the mechanically treated fibers that could also serve as defects in the matrix. Both of these defects would also be distributed very unevenly, therefore it looks like it looks like a possible explanation. A somewhat similar mechanism could be involved in composites with corroded fibers. For example, all composites made with HNO_3_ treated fibers acquired a brownish red color due to the corrosion products, but the corrosion products did not adhere to the surface of the fibers.

It is also interesting to note that the use of somewhat corroded fibers could also be beneficial as a result of mechanical interlocking, but the corrosion products and the leftover brass could serve as a relatively weaker link between the fiber and the matrix at times. Additionally, there is still a relative danger of brass dissolution in the case of HCl treated fibers. Bothoth HNO_3_ and HCl treated fibers could break when pulled out because of the corrosion, but there is no evidence behind this, even though the flexural strength of composites with fibers treated with HNO_3_ could also indicate this. This is also supported by the results regarding compressive strength values.

**Figure 9:**
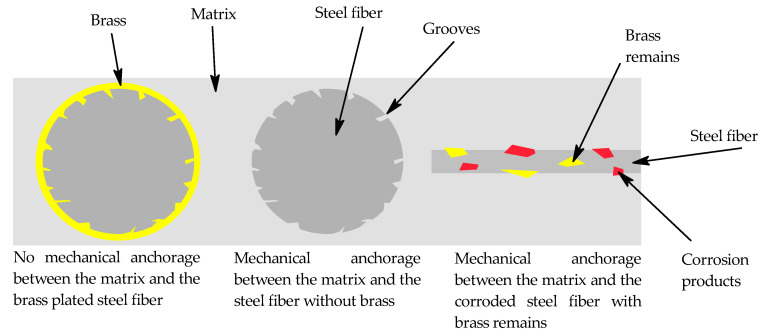
Scheme of possible mechanisms encountered regarding the steel fiber-matrix interface.

It is clear from all of the results presented above that the main mechanism with respect to the increase in flexural strength of composites with fibers treated in NH_3_/H_2_O_2_ is mechanical interlocking due to the formations on the surface of fibers treated in this solution. All the mechanisms discussed below are illustrated in a scheme in Figure 9. The removal of brass reveals the grooves beneath the brass on the surface of the fiber so that the matrix can penetrate them and physically secure the fiber in place. It also makes the fiber more resistant to being pulled out of the matrix. Some tiny components of the mix, such as micro silica, could also embed themselves into these grooves and serve as resistance to pulling from the fiber out of the matrix if they were able to scratch the fibers. The brass could not be removed as a whole, as seen in Figure 6, so the grooves could have been made deeper so that the overall effect of the brass removal would be greater. Mechanical interlocking regarding the remains of the brass or corrosion products on the fibers could also be considered, but this would rather serve as a weaker link between the fiber and the matrix than the grooves on the original surface of the steel fibers.

## 4. Conclusions

There are few conclusions that can be drawn from results above.

1. The majority of brass can be removed by highly oxidizing solutions, such as NH_3_/H_2_O_2,_ but there will always be some Cu left on the surface. The use of other chemicals such as (NH_4_)_2_CO_3_ could also potentially work, but their composition could not be optimized in this investigation because these solutions had never before been used in such a manner, so the optimization of these solutions was not the main goal.
2. As proposed, mechanical treatment of steel fibers for brass removal that could potentially be practical for modification of a large amount of fibers were also tried but it was not successful in terms of brass removal and it failed to achieve mechanical interlocking between steel fiber and matrix.
3. The brass coating on the surface of the steel fibers is relatively smooth and uneven in terms of elemental composition. Iron can even be detected by means of EDS analysis in some places. The brass covers grooves present on the original steel fiber, thus inhibiting mechanical interlocking between the steel fibers and matrix. This is the reason why uncoated fibers should always be used when available. Mechanical interlocking between steel fibers and matrix can be achieved by removal of brass or surface modification by relatively simple chemical solutions.
4. There is no major impact on the slump flow with respect to the brass coating of steel fibers or their surface modification, but attention should always be paid to particles such as metal dust or corrosion products in the matrix that could possibly play a role in decreasing slump flow.
5. No brass dissolution was observed under given conditions, but there is still the possibility of brass dissolution in other types of mix compositions, but attention should be paid to mentioning all variables regarding the possibility of corrosion.
6. The mechanical interlocking between the steel fiber and the matrix results in an increase of the flexural strength in terms of brass removal and incorporation of the matrix into the grooves present on the original fiber. Mechanical interlocking could also occur between the steel fiber and the matrix if there are corrosion products present on the fiber. The authors of this search believe that this effect can be applied to other mix compositions, but readers should always keep in mind that Aalborg white cement was used with high amounts of C_3_S and C_2_S and minimal amounts of C_3_A, so the effect of mechanical interlocking could be slightly different when other types of cement are used.

## Figures and Tables

**Table 1:** Applied chemicals, concentrations and experiment durations of fibers surface treatment.

Treatment Annotation	Applied Chemicals and Their Concentrations	Experiment Duration
NH_3_/H_2_O_2_	NH_3_ and H_2_O_2_ 3:1 (*v*/*v*)	Nearly instant
HNO_3_/KOH	Conc. HNO_3_ + KOH pH > 10	30 min in HNO_3_ + 15 min in KOH
HCl	HCl	15 min
(NH_4_)_2_CO_3_	(NH_4_)_2_CO_3_ 30 g/L + NH_3_	5 h
NH_4_Cl	NH_4_Cl 100 g/L + NH_3_	5 h
H_2_SO_4_	Conc. H_2_SO_4_	30 min

**Table 2:** Quality, supplier and origin of used chemicals.

Chemical	Quality, Supplier and Origin
NH_3_	For analysis, Penta, CZ
HNO_3_	
(NH_4_)_2_CO_3_	
NH_4_Cl	
H_2_SO_4_	
H_2_O_2_	
HCl	For analysis, MikroCHEM, SK
KOH	Technical quality 99%, Fichema, CZ
Ca (HCOO)_2_	Technical quality 98%, CHEMlogistics, CZ
Mesh 40 synthetic corundum	>95%, Abranova, CZ

**Table 3:** Raw materials used for composite fabrication.

Composite Constituents	Weight [g]
Fine sand according to CSN EN 196-1 (Filtracni pisky Chlum, CZ)	1980
Micronized sand ST-2 (Sklopisek Strelec, CZ)	135
Micro-dorsilit 110 (Dorfner, D)	405
CEM I 52.5 R -SR 5 white (Aalborg Portland, DE)	864
Silica fume RW Füller-Q (Elkem, D)	216
Steel fibers 12.5 × 0.2 mm (KrampeHarex, D)	300
Potassium sulphate (pure, Penta, CZ)	3
Potassium formate (synthesized)	34.4
	**Volume [mL]**
Superplasticizer MasterGlenium ACE 4446 (BASF, D)	45
Demineralized water	278

**Table 4:** XRF results.

Treatment Method	Cu [%]	Zn [%]	S [%]
Brass coated steel fibers	4.92	2.28	0.07
NH_3_ and H_2_O_2_ 3:1 (*v*/*v*)	0.05	0.00	0.08
Conc. HNO_3_ + KOH pH > 10	0.04	0.05	0.09
(NH_4_)_2_CO_3_ 30 g/L + NH_3_	0.71	0.32	0.08
NH_4_Cl 100 g/L + NH_3_	1.21	0.51	0.08
HCl	2.69	0.84	0.09
Conc. H_2_SO_4_	4.04	1.30	2.45
Mechanical treatment	3.39	1.36	0.09

**Table 5:** Average slump flow of fabricated composites.

Type of Treated Fibers in Composite	Spread Diameter
NH_3_/H_2_O_2_	188 mm
HNO_3_	184 mm
HCl	180 mm
Mechanical treatment	172 mm
